# Accumulation, Directional Delivery and Release of Nanoparticles along a Nanofiber

**DOI:** 10.3390/molecules27103312

**Published:** 2022-05-21

**Authors:** Mingcong Wen, Benjun Yao, Shun Yuan, Hongxiang Lei

**Affiliations:** School of Materials Science and Engineering, Nanotechnology Research Center, State Key Laboratory of Optoelectronic Materials and Technologies, Sun Yat-sen University, Guangzhou 510275, China; wenmc@mail2.sysu.edu.cn (M.W.); ybjsywfw@163.com (B.Y.); yuansh9@mail2.sysu.edu.cn (S.Y.)

**Keywords:** accumulation of nanoparticles, directional delivery, release, optical nanofiber

## Abstract

Controllably accumulating and delivering nanoparticles (NPs) into specific locations are a central theme of nano-engineering and important for targeted therapy or bacteria removal. Here we present a technique allowing bidirectional accumulation, directional delivery and release of nanoparticles through two 980-nm-wavelength counter-propagating evanescent waves in an optical nanofiber (NF). Using 713-nm-diameter polystyrene NPs suspension and an 890-nm-diameter NF as an example, we experimentally and theoretically demonstrate that the NPs delivered along the NF surface in opposite directions are accumulated into the region where the scattering loss of the NPs is maximum, and about 90% of the incident optical field from both ends of the NF can be coupled into the region. Moreover, the accumulation region can be controlled by altering the incident optical power ratio of the two counter-propagating laser beams, while the accumulated NPs can be delivered and then released into the specific locations by turning off the two lasers.

## 1. Introduction

Nanoparticle manipulation, especially massive trapping and directional delivery, holds the key to bionanotechnology [1,2]. Many methods have been proposed to manipulate micro/nono particles, such as electrical [3], magnetic [4], hydrodynamic [5] and optical [6] force. Since Arthur Ashkin used two counter-propagating focused beams to trap particles [7], optical forces have emerged as an efficient, non-contact and non-invasive manipulation of particles. However, conventional optical tweezers (COTs) are difficult for trapping nanoscale objects because they are subject to the optical diffraction limit and the trapping force reduces dramatically with the decreasing particle size [2,8]. Although the optical trapping force can be enhanced to manipulate smaller particles by simply increasing the laser power, this improvement is very limited and brings about irreversible photothermal damage [9]. Stable manipulation of nanoparticles can be achieved by increasing the “sharpness” of the optical intensity gradient [10,11] or through local amplification of the optical field [12,13]. Over the past few decades, in order to break the diffraction limit of the COTs, many nanotweezers based on near-field optics have been proposed, including slot waveguide [10,14], photonic crystal [15,16], plasmonic [17,18,19], optical fiber [20,21,22,23] and other [24,25,26] nanotweezers. Among these, with the advantages of longer delivery range and smaller couple loss, optical nanofibe is a more flexible and convenient tool for near-field optical manipulation in very narrow spaces [27,28,29]. When a laser beam is coupled into the nanofiber, the evanescent field leaking from the nanofiber decays exponentially along the axis perpendicular to the nanofiber surface, which produces a strong optical gradient. Therefore, particles interacting with the evanescent field can be trapped on the nanofiber surface by the strong optical gradient force and then delivered forward along the surface by the optical scattering force. Peer works about the targeted accumulation and release of massive nanoparticles using a defect-decorated optical nanofiber have been reported [30], but this method needs high precision for decorating defects in the nanofiber. 

Previously, we experimentally demonstrated that a nanofiber illuminated by two counter-propagating beams could stably trap one or several nanoparticles and controllably position them [31]. In addition, two counter-propagating beams along a bio-conveyor belt were used to stably trap and bidirectionally transport nanoparticles and biological cells [32]. This motivated us to accumulate massive nanoparticles, deliver and then release them into specific locations using an optical nanofiber with the advantages of easy fabrication and high flexibility, which will bring a potential application in biomedical and chemical fields such as sample analysis and preparation in a microdevices or chip, targeted therapy and bacteria removal. Therefore, in this work, using a 713 nm-diameter polystyrene nanoparticles suspension and an 890 nm-diameter NF as an example, we experimentally demonstrate a controlled bidirectional accumulation, directional delivery and release of nanoparticles (NPs) through two 980 nm-wavelength counter-propagating evanescent waves in the optical nanofiber (NF).

## 2. Experiments

**Figure 1:**
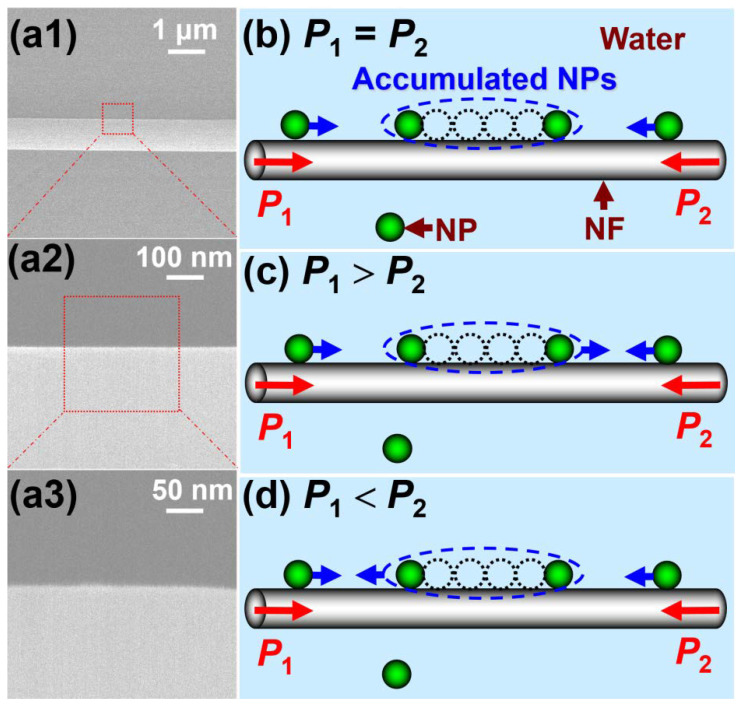
**Scanning electron microscope (SEM) image of the nanofiber and schematic of accumulation and delivery of nanoparticles (NPs) along a nanofiber (NF).** (**a1**–**a3**) SEM images of the nanofiber with magnifications of 10,000 (**a1**), 100,000 (**a2**) and 200,000 (**a3**). (**b**) When *P*_1_ = *P*_2_, the accumulated NPs will keep stationary on the NF. When *P*_1_ < *P*_2_ and *P*_1_ > *P*_2_, the accumulated NPs will be delivered to the right (**c**) and left (**d**) side of the NF, respectively.

Figure 1 shows the principle of the experiment. An 890 nm-diameter NF, which was drawn from a commercial single-mode optical fiber by the flame heating technique, was immersed in stationary suspension. Figure 1(a1–a3) shows the SEM images of the nanofiber with magnifications of 10,000, 100,000 and 200,000, respectively, which present good uniformity and sidewall smoothness. The average sidewall root-mean-square roughness is estimated to be about 0.5 nm. The suspensions were prepared by diluting 713 nm diameter polystyrene NPs into deionized water with the assistance of ultrasonics for 30 s (volume ratio of particles to water ~1:1000). Two laser beams with optical powers of *P*_1_ and *P*_2_ outputted from two pigtailed diode lasers with 980-nm wavelength were launched into the NF in the opposite directions. Here, choosing this wavelength was mainly because it can help to obtain a strong evanescent wave outside the surface of the NF and it has a low light absorption for most living matter. The NF, NPs and water are almost transparent to the wavelength of 980 nm, so absorption loss is ignored. For an ideal case, the trapped NPs can be delivered along the surface of the NF in two controlled directions or positioned on the surface of the NF by altering the incident optical power ratio [30,31]. Actually, the scattering of the NPs on the fiber leads to an additional optical loss, which affects the delivering velocities of the NPs. In general, keeping the incident optical power unchanged, the intensity of the evanescent wave will gradually and slowly decrease, which makes the delivering velocities decrease accordingly. Additionally, NPs that are not strictly uniform in sizes have non-uniform delivering velocities. Specifically, the delivering velocities for larger NPs are higher than those for smaller NPs [27]. Thus, the above two factors caused the non-uniform delivering velocities of NPs in the experiment, which will easily make the trapped NPs form a short chain. The short chain can act as a larger NP, which has a larger delivering velocity and could continue to chase the other “small” moving NPs. By repeatedly altering the incident optical power ratio, more and more trapped NPs will form a longer chain, where the two counter-propagating laser beams are almost coupled into the row of NPs and the scattering loss of the NPs reaches a maximum. That is, the transmission of the light will be strongly suppressed at the other side of the chain. As a result, the position forms an accumulation region and the NPs delivered along the NF in the opposite directions are accumulated into the region, as shown in Figure 1a–c. Moreover, the accumulation region can be controlled by altering the incident optical power ratio (*η*) of the two incident laser beams. Specifically, when *η* = *P*_2_/*P*_1_ = 1 (here *P*_1_, *P*_2_ denotes the incident optical power of the left and right ends of the NF, respectively), the accumulation region keeps stationary on the NF, as shown in Figure 1a. When *η* < 1, the accumulation region will be delivered to the right side of the NF, as shown in Figure 1b. When *η* > 1, the accumulation region will be delivered to the opposite (left) side of the NF, as shown in Figure 1c. That is, the motion direction of NPs at the accumulation region on NF also exhibit the opposite direction with an increasing *η*, which can be further explained in the latter simulation. According to this analysis, we can achieve the controlled accumulation of the NPs, directional delivery and release to the specific locations by turning off the two lasers. To indicate the delivery direction, we defined the direction to right as the positive direction. Here, it should be pointed out that, repeatedly altering the incident optical power ratio in the forming process of a longer chain was mainly to ensure the accumulating process of the long chain was within the same view field and improved the accumulating efficiency. Alternatively, the longer chain could also be formed at one incident optical power ratio, but it could be subjected to the limit of the maximum accumulated number because of the weak optical gradient force. Moreover, the method needs to adjust the microscope stage to ensure it is within the view of the microscope, which can easily cause environmental disturbance and then decrease the accumulating efficiency.

**Figure 2:**
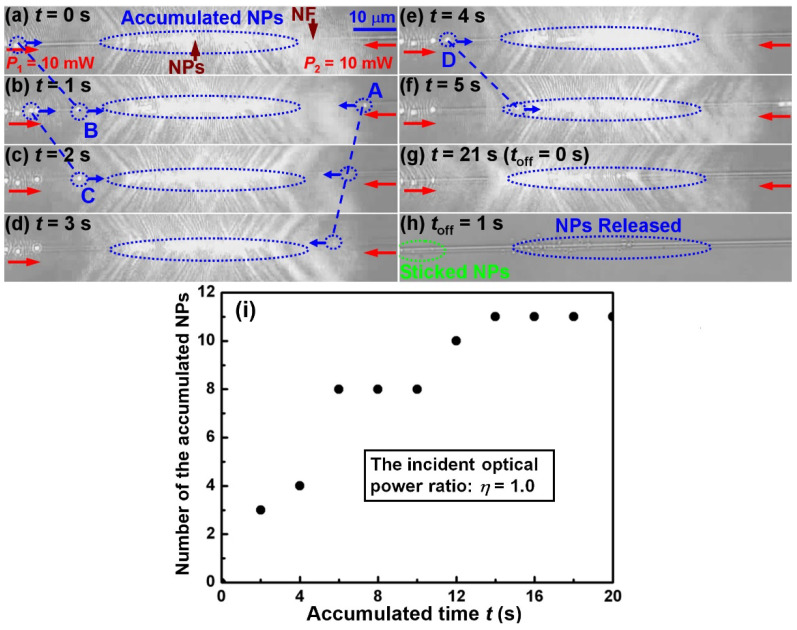
**Bidirectional accumulation of NPs and release.** (**a**–**f**) Accumulation of NPs (labeled as A, B, C, D) for *t* = 0 to 5 s with the same incident optical power (*P*_1_ = *P*_2_ = 10 mW, i.e., *η* = *P*_2_/*P*_1_ = 1) at both ends of the NF. (**g**) Microscope image of the laser just before being turned off (i.e., *t*_off_ = 0 s, corresponding t = 21 s). (**h**) Release of the accumulated NPs at *t*_off_ = 1 s. Detailed bidirectional accumulation and directional release process from *t* = 0 to 22 s is shown in Media S1. (**i**) Number of the accumulated NPs in the corresponding accumulated time.

A series of experiments were performed using the model shown in Figure 1. As an example, Figure 2a–f shows the consecutive bidirectional accumulation images of NPs with the same incident optical power (*P*_1_ = *P*_2_ = 10 mW, i.e., *η* = 1) at both ends of the NF. At the beginning of the experimental recording (*t* = 0 s), about 19 NPs were accumulated in the accumulation region (blue dashed line circle), where the delivered NPs from two opposite directions accumulated (Figure 2a). For example, from *t* = 0 to 5 s, NPs (labelled as A, B, C, D) were accumulated (Figure 2a–f). The delivering distances of NPs A, B, C and D were 7.5, 14.6, 11.2, and 16.2 μm, respectively. The estimated average delivering velocities on both sides of the accumulation region were −3.8 and 14.0 μm/s, respectively. Here, the variation of the trapping position above the NF affects the delivering velocities of NPs and thus leads to the difference between the two velocities. We further found that in the whole bidirectional accumulation process, the accumulation region remained at the same locations, mainly because of the same incident optical power at both ends of the NF. Therefore, if we turn off the laser in this case, the accumulated NPs will be released to the specific locations. Figure 2g shows the microscope image of the laser just before being turned off (i.e., *t*_off_ = 0 s, corresponding *t* = 21 s). There were about 30 nanoparticles in the accumulation region, and their size was about 18.0 × 1.0 μm. In the next moment, the 30 NPs were released. Figure 2h shows the microscope image of the released NPs at *t*_off_ = 1 s. Because of the unsmooth surface, several NPs (green dashed line circle) were stuck on the NF in the accumulation process. The detailed bidirectional accumulation and directional release process with the same incident optical power *P*_1_ and *P*_2_ from *t* = 0 to 22 s is shown in Supplementary Materials Media S1. From the Media, we can also obtain the number of the accumulated NPs in the corresponding accumulated time, as shown in Figure 2I. It can be seen that the number of the accumulated NPs become more and more with increasing accumulated time. Typically, 11 NPs were accumulated into the accumulation region in 21 s.

**Figure 3:**
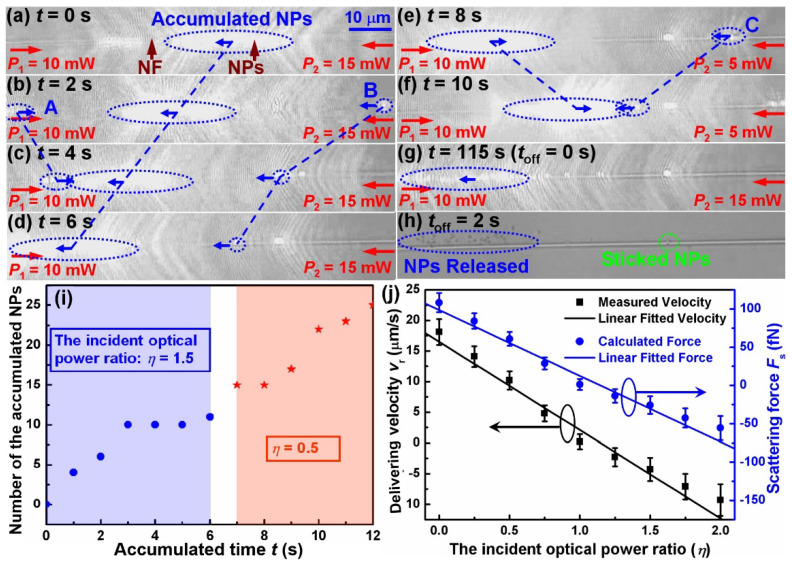
**Bidirectional accumulation of NPs, deliveryand release.** (**a**–**d**) Accumulation of NPs (labeled as A, B) and delivery with *P*_1_ = 10 mW and *P*_2_ = 15 mW (i.e., *η* = 1.5). In this case, the accumulated NPs (blue dashed line circle, about 20 NPs that were accumulated at *t* = 0 s) were delivered in the negative direction (from right to left). (**e**,**f**) Accumulation of NPs (labelled as C) and delivery with *P*_1_ = 10 mW and *P*_2_ = 5 mW (i.e., *η* = 0.5). In this case, the accumulated NPs were delivered in the positive direction. (**g**) Microscope image of the laser just before being turned off (i.e., *t*_off_ = 0 s, corresponding t = 115 s). (**h**) Release of the accumulated NPs at *t*_off_ = 2 s. Detailed bidirectional accumulation, directional delivery and release process with the different incident optical power ratios from *t* = 0 to 117 s is shown in Media S2. (**i**) Number of the accumulated NPs with the corresponding accumulated time from *t* = 0 to 12 s. (**j**) Delivering velocity (*v*_r_) of NPs in the accumulation region and the corresponding optical scattering force acting on them as a function of *η* (keeping *P*_1_ = 10 mW unchanged).

Remaining the incident optical power at the left end of the NF at *P*_1_ = 10 mW unchanged, while altering the optical power *P*_2_ at the right end of the NF, we found that the accumulation region can be movable and its delivering direction and velocity can be controlled. Moreover, the trapped NPs at both sides can yet be delivered and then accumulated to the region. For examples, when increasing the incident optical power at the right end of the NF to *P*_2_ = 15 mW (i.e., *η* = 1.5), the accumulated NPs (blue dashed line circle, about 20 NPs have been accumulated at *t* = 0 s) are delivered to the left side, as shown in Figure 3a–d. Moreover, the trapped NPs (A, B as examples) are delivered and then accumulated in the region. The delivering distances of NPs A, B, and the accumulated NPs were 9.6, 34.7 and 25.8 μm, respectively, and the estimated average delivering velocities are 4.8, −8.7 and −4.3 μm/s, respectively. On the contrary, when decreasing the incident optical power at the right end of the NF to *P*_2_ = 5 mW (i.e., *η* = 0.5) at *t* = 7 s, the accumulated NPs are delivered to the right side. As an example, Figure 3e,f shows the phenomenon at *t* = 8 and 10 s, respectively. Moreover, the trapped NPs (C as an example) at the right side of the accumulated NPs region are delivered to the left under actions of the optical scattering force from the right laser beams with an optical power of 5 mW. Then it was accumulated to the region. The delivering distance of NPs C and the accumulated NPs are 23.0 and 20.4 μm, respectively, and the estimated average delivering velocities are −11.5 and 10.2 μm/s, respectively. Similar to the above experiment, the accumulated NPs will also be released to the specific locations by turning off the laser. Figure 3g shows the microscope image of the laser just before being turned off (i.e., *t*_off_ = 0 s, corresponding *t* = 115 s). There are 116 nanoparticles in the accumulation region, and the corresponding size of the accumulation region is about 20.0 × 4.0 μm. In the next moment, the 116 NPs will be released. Figure 3h shows the microscope image of the released NPs at *t*_off_ = 2 s. In the whole accumulation process, two NPs (green dashed line circle) were stuck on the NF. Detailed bidirectional accumulation, directional delivery and release process with the different incident optical power ratio from *t* = 0 to 117 s is shown in Media S2. From the Media, we can also obtain the number of the accumulated NPs in the corresponding accumulated time. As an example, Figure 3I shows the number of the accumulated NPs with the accumulated time from *t* = 0 to 12 s. The blue region represents the incident optical power ratio *η* = 1.5 from *t* = 0 to 6 s, and the red region represents *η* = 0.5 from *t* = 7 to 12 s. It can be seen that the number of the accumulated NPs increases with increasing accumulated time. Typically, 24 NPs were accumulated into the accumulation region in 12 s. Then, by repeatedly changing the incident optical power *P*_2_, the accumulation region can be propelled back and forth on the screen of the Media and the accumulated NPs gradually increase. In the whole accumulated time of 115 s, about 96 NPs were accumulated.

To further investigate the influence of the incident optical power ratio *η* on the NPs’ accumulation, the delivering velocity (*v*_r_) of NPs in the accumulation region was measured as a function of *η* (keeping *P*_1_ = 10 mW unchanged), as shown in Figure 3J. It can be seen that, with the increasing *η*, the *v*_r_ shows a linearly descending trend with a fitting equation of *v*_r_ = –14.4*η* + 16.5. Moreover, at *η* = 1, *v*_r_ = 0 μm/s, the accumulated NPs were halted at the corresponding position of the NF. When *η* < 1, *v*_r_ > 0, the accumulated NPs were delivered toward the right side of the NF. When *η* > 1, *v*_r_ < 0 and the accumulated NPs were delivered toward the left side of the NF. Within the length of the nanofiber (a few millimetres in this work), the delivery would not stop unless *η* = 1. Additionally, we estimated the corresponding optical scattering force by using Stokes law *F* = 6π*rμν*_r_, where *r* = 356.5 nm is the radius of PS particle, *μ* = 8.9 × 10^−4^ Pa·s is the dynamic viscosity of water at room temperature. The estimated results are also shown in Figure 3J. It can be seen that the scattering force also decreased linearly with increasing *η*, with a linear relationship of *F*_s_ = –86.2*η* + 98.5. Here, it should be noted that Figure 2a–g, Figure 3a–g and the corresponding media looked like they suffered from strong over-exposure, which was mainly because of the strong scattering light of the accumulated NPs and a high response of microscope CCD to the light with 980 nm wavelength.

## 3. Theoretical Analysis and Discussion

**Figure 4:**
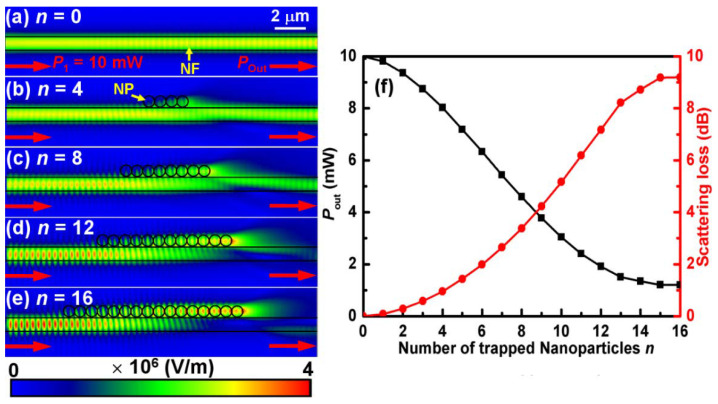
**Simulation and calculation results for the scattering characteristics of the NPs chain on the NF.** (**a**–**e**) Simulated optical field distributions with the different number (*n*) of the trapped NPs in the formed chain and the same incident optical power (*P*_1_ = 10 mW). (**f**) The output optical power *P*_out_ and the calculated scattering losses *l* with different *n*.

To explain the above phenomena, three-dimensional (3D) finite-difference time-domain (FDTD) simulations were performed by setting the refractive indices of the polystyrene NPs, water and NF to be 1.573, 1.33 and 1.445, respectively. The gap between the NF surface and the polystyrene particle was set as 10 nm, which is consistent with the Debye length [28]. Firstly, we investigated the scattering characteristics of the NPs’ chain on the NF. Figure 4a–e, as an example, shows the simulated optical field distributions with the different number (*n*) of the trapped NPs in the formed chain and the same incident optical power (*P*_1_ = 10 mW). The measured optical loss is extremely small (about 0.15 dB), which has been neglected. It can be seen that, with an increase of *n*, more and more optical fields are coupled into the NPs chain and the outputted optical field become weaker, especially when *n* = 16, where there is almost no light on the output end of the NF, as shown in Figure 4e. To further study the outputted optical power (*P*_out_) and the induced scattering losses (*l* = −10log(*P*_out_/*P*_1_) dB) with different *n*, a series of simulations were carried out. Figure 4f shows the *P*_out_ and the calculated scattering losses *l* with different *n*. It can be seen that the *P*_out_ decreases with increasing *n* and reaches a minimum value (~1.1 mW) at *n* ≥ 15. On the contrary, the scattering losses *l* exhibits an ascending trend with increasing *n* and reaches a maximum value (~9.1 dB) at *n* ≥ 15. According to the above analysis, it is concluded that when *n* ≥ 15, about 90% of the incident optical fields are coupled into the NPs chain, and thus only the NPs at the left side of the chain can be trapped and then delivered along the NF till reach the chain region. Here, it should be pointed out that the simulated results in Figure 4 do not mean that the maximum number of accumulated NPs is theoretically 15 at the optical power of 10 mW, but mean that when the number is larger than or equal to 15, most of light is coupled into the accumulated NPs region. In this case, the transmission of the light is strongly suppressed at the right side of the region and more trapped NPs on the nanofiber from the left side of the accumulated NPs region would be delivered into the accumulated region by the optical scattering forces to the right. The accumulated NPs, as bigger particles, would also be trapped and delivered along the nanofiber with an action of the optical force. The accumulation would be stopped until the optical gradient force acted on the accumulated NPs became too weak to trap them. But, when another laser beam was launched into the other end of the fiber, more NPs would be accumulated into the accumulated region because of larger optical gradient force acting on them. After all, the gradient force could be composited and become stronger (as shown in the following simulation).

**Figure 5:**
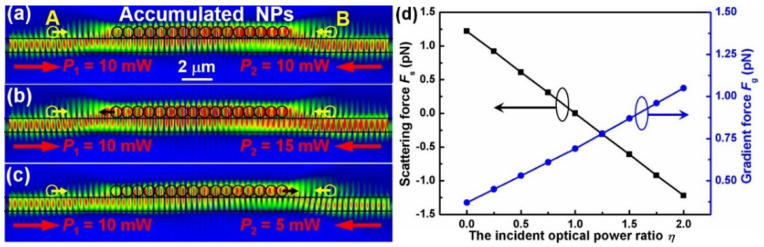
**Simulation and calculation results for accumulation of the NPs and delivery along NF (keeping *P*_1_ = 10 mW unchanged).** (**a**–**c**) Simulated distributions of the optical field for three different input optical powers *P*_2_ = 10 (**a**), 15 (**b**) and 5 mW (**c**). (**d**) Calculated forces *F*_s_ and *F*_g_ of the accumulated NPs in the accumulation region with different incident optical power ratios *η* (keeping *P*_1_ = 10 mW unchanged).

Next, taking *n* = 16 as an example, we analyzed the bidirectional accumulation phenomena by simulating the optical field distribution with two counter-propagating laser beams. Keeping the incident optical power at the left end of the NF at *P*_1_ = 10 mW unchanged, the simulated distributions of the optical field for three different input optical powers (*P*_2_ = 10, 15, 5 mW) at the right end of NF are shown in Figure 5a–c. It can be seen that the optical fields at both sides of the NPs chain are not superposed with each other but coupled into the NPs chain and then superposed there. Moreover, a proportion of the optical fields propagate as evanescent waves outside the NF, which act on the NPs near the NF in the form of the gradient force (*F*_g_) and the scattering force (*F*_s_). Here, the *F*_g_ will trap the NPs to the NF surface, and the *F*_s_ will deliver NPs along the direction of the light propagation. Therefore, under the action of the *F*_g_ and the *F*_s_, the NPs at both sides of the NPs chain are trapped and then delivered towards the accumulation region. At the accumulation region, taking the accumulated NPs as a larger particle, the *F*_g_ acted on the larger particle will be composited and become stronger with increasing laser power at the other end of the nanofiber, which makes the trapping of the larger particle more stable. In contrast, the *F*_s_ will be partially counteracted and exhibit a descending trend with an increasing laser power at the other end of the nanofiber, which makes the delivering velocity of the larger particle be gradually decreased. When the increasing laser power exceeds the power of the fixed end, the direction of the *F*_s_ will be changed, which causes a corresponding change in the delivering direction of the larger particle. Therefore, the delivering direction and velocity of the larger particle will be controllable. To numerically show the above performance, as an example, both the optical forces exerted on the particles A, B and the accumulated NPs were calculated by taking the integral of the Maxwell stress tensor around the particles [32]. To indicate the direction of the optical gradient force and the optical scattering force, we defined the direction down and the direction to right as the positive direction, respectively. Because of the unchanged incident optical power *P*_1_ at the left end of the NF, the calculated *F*_g_/*F*_s_ basically kept the invariable at ~ 0.34/1.23 pN (pico Newton) for particle A, as shown in Figure 5a–c. For particle B, the calculated *F*_g_/ *F*_s_ were 0.34/−1.23, 0.51/−1.85 and 0.17/−0.62 pN, respectively. As a result, we could achieve the bidirectional accumulation of the NPs. 

For the accumulated NPs, the calculated *F*_g_/*F*_s_ were 0.69/0, 0.87/−0.61 and 0.53/0.61 pN, respectively. In more details, Figure 5d shows the calculated forces *F*_s_ and *F*_g_ of the accumulated NPs in the accumulation region with different incident optical power ratios *η* (keeping *P*_1_ = 10 mW unchanged). It can be seen that, the force *F*_g_ increases with increasing *η*, which is owed to the composition of energy density along this direction and makes the trapping of the larger particle more stable. The force *F*_s_ exhibits a descending trend with increasing *η*, and the direction of *F*_s_ is changeable. Therefore, the accumulation region can also be bidirectionally controlled, and thus, the accumulated NPs could be directionally released to the specific locations by turning off the two lasers, which is consistent with the former experimental results and will bring potential applications in targeted therapy and bacteria removal. 

The difference of *F*_s_ between the calculated and experimental results is mainly induced by the different number of NPs in the accumulated region, the different scattering loss of the NPs on the NF, the variation of trapping position above the NF and environmental disturbances in the experiment. The first factor, in particular, has the greatest impact. Here, it should be pointed out that 16 was not theoretically the maximum number in the accumulated region. It means that when the number is 16, most of the light from two counter-propagating laser beams is coupled into the NPs chain in the experiment. More trapped NPs on the nanofiber from two sides of the accumulated region could be delivered into the region, which would increase the number of accumulated NPs. It is in agreement with the above experimental results. Additionally, from the simulated results, the propagating laser beams could be coupled into the accumulated NPs chain, which could be another optical nanowaveguide and more NPs might be trapped on the upper surface of the chain [32], which might cause an additional accumulation of NPs in the accumulation region. Moreover, the Brownian motion of NPs and environmental disturbances in the experiment are also two factors affecting the chain structure. Thus, not a stable long chain but an accumulation of NPs was formed in the accumulation region, which is faintly visible in Figure 2 and Figure 3 and the corresponding Media.

## 4. Conclusions

We have theoretically and experimentally demonstrated particles’ accumulation and delivery to special locations using an optical nanofiber. By launching two counter-propagating 980 nm wavelength laser beams into an 890 nm diameter nanofiber, 713 nm diameter polystyrene particles were bidirectionally accumulated into the region and the accumulation region were controlled by altering the incident optical power ratio in the NF. Moreover, the accumulated NPs were released into specific locations by turning off the two lasers at the corresponding moment. The results are expected to find applications in the biomedical and chemical fields.

## Data Availability

Not applicable.

## Supplementary Materials

The following supporting information can be downloaded at: https://www.mdpi.com/article/10.3390/molecules27103312/s1, Media S1: Detailed bidirectional accumulation and directional release process with the same incident optical power (*P*_1_ = *P*_2_ = 10 mW, i.e., *η* = 1) at both ends of the NF. Media S2: Detailed bidirectional accumulation, directional delivery and release process with the different incident optical power ratio from *t* = 0 to 117 s.

## Sample Availability

Polystyrene nanoparticles were purchased from Huake Sphere Scientific Co., Ltd. Wuhan, China.

## References

[1] Gao D., Ding W., Nieto-Vesperinas M., Ding X., Rahman M., Zhang T., Lim C., Qiu C.-W. (2017). Optical manipulation from the microscale to the nanoscale: Fundamentals, advances and prospects. Light Sci. Appl..

[2] Xin H., Li Y., Liu Y., Zhang Y., Xiao Y., Li B. (2020). Optical forces: From fundamental to biological applications. Adv. Mater..

[3] Yazbeck R., Alibakhshi M.A., Von Schoppe J., Ekinci K.L., Duan C. (2019). Characterization and manipulation of single nanoparticles using a nanopore-based electrokinetic tweezer. Nanoscale.

[4] Cao Q., Fan Q., Chen Q., Liu C., Han X., Li L. (2020). Recent advances in manipulation of micro- and nano-objects with magnetic fields at small scales. Mater. Horiz..

[5] Volpe A., Gaudiuso C., Ancona A. (2019). Sorting of particles using inertial focusing and laminar vortex technology: A review. Micromachines.

[6] Shilkin D.A., Lyubin E.V., Shcherbakov M.R., Lapine M., Fedyanin A.A. (2017). Directional optical sorting of silicon sanoparticles. ACS Photonics.

[7] Ashkin A. (1970). Acceleration and trapping of particles by radiation pressure. Phys. Rev. Lett..

[8] Crozier K.B. (2019). Quo vadis, plasmonic optical tweezers?. Light Sci. Appl..

[9] Blázquez-Castro A. (2019). Optical tweezers: Phototoxicity and thermal stress in cells and biomolecules. Micromachines.

[10] Yang A.H., Moore S.D., Schmidt B.S., Klug M., Lipson M., Erickson D. (2009). Erickson, Optical manipulation of nanoparticles and biomolecules in sub-wavelength slot waveguides. Nature.

[11] Cao T., Wang Z.M., Mao L.B. (2022). Reconfigurable label-free shape-sieving of submicron particles in paired chalcogenide waveguides. Nanoscale.

[12] Sahafi M., Habibzadeh-Sharif A. (2019). All-optical trapping, relocation and manipulation of nanoparticles using SOI ring resonators. J. Opt. Soc. Am. B.

[13] Pin C., Magno G., Ecarnot A., Picard E., Hadji E., Yam V., De Fornel F., Dagens B., Cluzel B. (2020). Seven at one blow: Particle cluster stability in a single plasmonic trap on a silicon waveguide. ACS. Photonics.

[14] Stievater T.H., Kozak D.A., Pruessner M.W., Mahon R., Park D., Rabinovich W.S., Fatemi F.K. (2016). Modal characterization of nanophotonic waveguides for atom trapping. Opt. Mater. Express.

[15] Yang D., Gao F., Cao Q.-T., Wang C., Ji Y., Xiao Y.-F. (2018). Single nanoparticle trapping based on on-chip nanoslotted nanobeam cavities. Photon. Res..

[16] Gao Y., Shi Y.C. (2019). Design of a single nanoparticle trapping device based on bow-tie-shaped photonic crystal nanobeam cavities. IEEE Photon. J..

[17] Ren Y., Chen Q., He M., Zhang X., Qi H., Yan Y. (2021). Plasmonic optical tweezers for particle manipulation: Principles, methods, and applications. ACS Nano.

[18] Peng X.L., Kotnala A., Rajeeva B.B., Wang M.A., Yao K., Bhatt N., Penley D., Zheng Y.B. (2021). Plasmonic nanotweezers and nanosensors for point-of-care application. Adv. Opt. Mater..

[19] Ghosh S., Ghosh A. (2018). Mobile nanotweezers for active colloidal manipulation. Sci. Robot..

[20] Li Y.C., Xin H.B., Zhao Y., Lei H.X., Zhang T.H., Ye H.P., Saenz J.J., Qiu C.W., Li B.J. (2018). Living nanospear for ner-field optical probing. ACS Nano.

[21] Li Y., Liu X., Li B. (2019). Single-cell biomagnifier for optical nanoscope and nanotweezers. Light Sci. Appl..

[22] Xin H., Li B. (2019). Fiber-based optical trapping and manipulation. Front. Optoelectron..

[23] Li Y., Xin H., Zhang Y., Li B. (2021). Optical fiber technologies for nanomanipulation and biodetection: A review. J. Lightw. Technol..

[24] Lin L., Wang M., Peng X., Lissek E.N., Mao Z., Scarabelli L., Adkins E., Coskun S., Unalan H.E., Korgel B.A. (2018). Opto-thermoelectric nanotweezers. Nat. Photonics.

[25] Conteduca D., Brunetti G., Pitruzzello G., Tragni F., Dholakia K., Krauss T.F., Ciminelli C. (2021). Exploring the limit of multiplexed near-field optical trapping. ACS Photonics.

[26] Xu Z., Crozier K.B. (2019). All-dielectric nanotweezers for trapping and observation of a single quantum dot. Opt. Exp..

[27] Xu L., Li Y., Li B. (2012). Size-dependent trapping and delivery of submicro-spheres using a submicrofibre. N. J. Phys..

[28] Xin H., Cheng C., Li B. (2013). Trapping and delivery of escherichia coli in a microfluidic channel using an optical nanofiber. Nanoscale.

[29] Zhao X.T., Zhao N., Shi Y., Xin H.B., Li B.J. (2020). Optical fiber tweezers: A versatile tool for optical tapping and manipulation. Micromachines.

[30] Xin H., Li B. (2011). Targeted delivery and controllable released of nanoparticles using a defect-decorated optical nanofiber. Opt. Express.

[31] Lei H., Xu C., Zhang Y., Li B. (2012). Bidirectional optical transportation and controllable positioning of nanoparticles using an optical nanofiber. Nanoscale.

[32] Liu X., Wu Y., Xu X., Li Y., Zhang Y., Li B. (2019). Bidirectional transport of nanoparticles and cells with a Bio-conveyor belt. Small.

